# Diurnal rhythms of vitamin D binding protein and total and free vitamin D metabolites

**DOI:** 10.1016/j.jsbmb.2017.07.015

**Published:** 2017-09

**Authors:** Kerry S. Jones, Jean Redmond, Anthony J. Fulford, Landing Jarjou, Bo Zhou, Ann Prentice, Inez Schoenmakers

**Affiliations:** a Medical Research Council Human Nutrition Research, Fulbourn Road, Cambridge CB1 9NL, United Kingdom; b MRC International Nutrition Group, London School of Hygiene & Tropical Medicine, Keppel Street, London WC1E 7HT, United Kingdom; c MRC Keneba, MRC Unit, Gambia; d Department of Public Health, Shenyang Medical College, Shenyang 110034, PR China

**Keywords:** DBP, vitamin D binding protein, DR, diurnal rhythm, CCV%, coefficient of cyclic variation%, 25(OH)D, 1,25(OH)_2_D, Fourier regression, Bioavailability, Africa, Diurnal

## Abstract

Vitamin D binding protein (DBP) concentration is known to influence the availability and bioactivity of vitamin D metabolites but its diurnal rhythm (DR), its inter-relationships with the DRs of vitamin D metabolites and its influence on free vitamin D metabolite concentrations are not well described.

The DRs of plasma total 25(OH)D, total 1,25(OH)_2_D, DBP, albumin and calculated free 25(OH)D and free 1,25(OH)_2_D were measured in men and women aged 60–75 years and resident in the UK (*n* 30), Gambia (*n* 31) and China (*n* 30) with differences in lifestyle, dietary intake and vitamin D status. Blood samples were collected every 4 h for 24 h and DRs statistically analysed with Fourier regression.

Gambians had significantly higher plasma concentrations of vitamin D metabolites and lower albumin concentration compared to the British and Chinese. Significant DRs were observed for all analytes and calculated free vitamin D metabolites (P < 0.01). The pattern of DRs was similar between countries. The magnitude of the DRs of free 1,25(OH)_2_D was attenuated compared to that of total 1,25(OH)_2_D whereas it was not different between total and free 25(OH)D. Relationships between the DRs were generally weak. There was no phase shift between 1,25(OH)_2_D and DBP with the strongest cross correlation at 0 h time lag (*r* = 0.15, P = < 0.001). In comparison, 25(OH)D correlated less well with DBP (1 h time lag, *r* = 0.07, P = 0.12).

These data demonstrate a relationship between the DRs of 1,25(OH)_2_D and DBP, possibly to maintain free 1,25(OH)_2_D concentrations. In contrast, the DRs of total and free 25(OH)D appeared to be less influenced by DBP, suggesting that DBP has comparatively less effect on 25(OH)D concentration and 25(OH)D availability. This work highlights the importance of standardisation in timing of sample collection particularly for the assessment of plasma protein concentrations.

## Highlights

• Plasma concentrations of DBP, albumin, 25(OH)D & 1,25(OH)_2_D exhibited significant diurnal rhythms (DR).
• DRs were similar in British, Gambian and Chinese men and women aged 60–75 years.
• The free 1,25(OH)_2_D DR was attenuated compared to that of total 1,25(OH)_2_D.
• The magnitude of the free 25(OH)D DR was not different to that of total 25(OH)D.

## 1 Introduction

Vitamin D is important in calcium, phosphate and bone metabolism and may be important in other aspects of human health. The total plasma concentration of the pro-hormone 25-hydroxyvitamin D (25(OH)D) is measured to determine vitamin D status. The majority of 25(OH)D in the circulation is bound to vitamin D binding protein (DBP) or albumin with only around 1% circulating in its free form. On the basis of the free hormone hypothesis it is this free fraction that has access to the majority of tissues, thus the concentration of free 25(OH)D may provide a marker of 25(OH)D availability to cells and tissues [1], [2]. Some tissues, including the kidney, internalise DBP-bound 25(OH)D via the plasma membrane transporter protein, megalin. The relative importance of bound and free 25(OH)D for different organ systems remains an area of active research. A stronger association between serum calcium, PTH, bone and vascular outcomes and inflammation with free 25(OH)D concentration are reported by some but not all studies (reviewed in [3], [4]). However, many of these studies were confounded by the use of particular assays [5]. In addition, 1,25(OH)_2_D, rather than 25(OH)D, is the biologically active vitamin D molecule that activates the vitamin D receptor (VDR) in cells. Like 25(OH)D, the majority of 1,25(OH)_2_D in the circulation is bound to DBP and some tissues internalise DBP-bound 1,25(OH)_2_D. Thus free 1,25(OH)_2_D concentration may provide better information on the systemic biological activity of 1,25(OH)_2_D than its total plasma concentration [6], [7].

There is pronounced 24 h variation or diurnal rhythm (DR) in the plasma concentrations of calcium and phosphate regulating hormones, in particular PTH which is the main regulator of systemic renal 1,25(OH)_2_D production [8], [9]. Reports of a DR in the plasma concentration of 1,25(OH)_2_D and other vitamin D metabolites are less consistent. This may be partly due to the potential influence of a simultaneous DR of DBP. The DRs of vitamin D metabolites and DBP and their inter-relationships may impact on the concentration and proportion of free or bound vitamin D metabolites available to tissues for internalisation. The influence of this variation will depend on the extent to which cells or tissues rely on the megalin-mediated or megalin–independent mechanisms for vitamin D uptake and availability to activate the VDR. Data on the DRs of 25(OH)D, 1,25(OH)_2_D and DBP are required to determine variations in the free fractions to understand vitamin D metabolism, particularly availability for extra-renal metabolism, and the potential importance of the timing of sample collections in research studies of clinical populations.

Here, combining novel data on 25(OH)D and DBP with data from a previous study of 1,25(OH)_2_D [9], we describe the DRs of plasma 25(OH)D, 1,25(OH)_2_D, DBP, albumin and free vitamin D metabolites. The data were derived from studies in three different ethnic groups that allowed exploration of the consistency of DRs in groups despite genetic and environmental differences that influence DBP subtype [10] and vitamin D status.

## 2 Methods

Descriptions of participants, study design and statistical analyses have been published previously in full [9] and are described in summary below.

### 2.1 Study location

The studies were conducted at three research centres: (1) MRC Human Nutrition Research, Cambridge UK, (2) MRC Keneba, The Gambia and (3) Shenyang Medical College, Shenyang, PR China. The characteristics of each population were described previously [9], [11], [12]. The British and Chinese studies were performed in winter when there is no cutaneous synthesis of vitamin D. The Gambia has two seasons, a ‘wet’ or ‘rainy’ season between June and October characterised by cloud and heavy rain and a ‘dry’ season characterised by largely clear skies and little, if any rainfall. This study was performed in the dry season during which there is expected to be little variation in UVB supply or potential UVB exposure. The study in each country was approved by their respective Ethics committee [9] and all participants provided written, informed consent. All research was performed in accordance with the Declaration of Helsinki.

### 2.2 Study participants

Participants were apparently healthy, free-living men and women aged 60 to 75 years recruited from the local community. Exclusion criteria were any known pathological disorder that may alter calcium or bone metabolism [11], haemoglobin < 10 g/dL and plasma creatinine > 115 μmol/L [9].

### 2.3 Study design

This was a secondary analysis of samples and data collected in a study designed to investigate differences in the DRs of PTH and bone metabolism markers [9]. The sample size of 15 individuals per sex from the original study was based on the ability to detect a 6% difference between the peak and nadir of PTH concentration (significance level of 5% and power of 80%) [9]. Participants were studied over a 24 h period and encouraged, as far as possible, to maintain normal activities and eating and sleeping routines. Sample collections were performed at standardized times at the participants’ homes and/or respective research centres as per the arrangements for each country [9]. Fasting blood samples were collected from all participants in the early morning. Subsequent blood samples were collected in the non-fasting state every 4 h. Participants were assigned to one of two groups whose blood collections were staggered by two hours and the data combined to derive a group level DR (see Section 2.5 Data analysis) [9].

### 2.4 Biochemical analysis

Plasma samples for biochemical analysis were transported from The Gambia and China on dry ice and, as with the UK samples, were stored at −80 °C until analysis. Plasma total 25(OH)D was measured by Liaison (Diasorin Ltd, Dartford, UK), total 1,25(OH)_2_D by radioimmunoassay (IDS Ltd, Tyne and Wear, UK), as described previously [9] and DBP by polyclonal antibody ELISA (Immunodiagnostik AG (Oxford Biosystems, Oxford, UK)) and albumin with an automated colorimetric method (Kone Lab 20i clinical chemistry, Thermo Scientific, Vantaa, Finland). All samples were measured in duplicate and analysis repeated if the CV was more than 10%. Assay performance was measured using kit and in-house controls and performance was in acceptable limits. External quality assurance was obtained through DEQAS (www.deqas.org) for plasma 1,25(OH)2D and 25(OH)D and traceable to NIST standards for 25(OH)D. This Diasorin Liaison assay in our lab has been calibrated against standardized LC–MS/MS reference measurement procedures developed by NIST and Ghent University. The relationship between the two methods is described by the equation: Diasorin Liaison = 1.023*LC–MS/MS–2.52 nmol/l (r^2^ = 0.8751, n = 186).

### 2.5 Data analysis

Data are presented as the mean (SD) or geometric mean (95% CI). For consistency with DR model parameters which are logged for analysis, baseline 25(OH)D, 1,25(OH)_2_D, DBP and albumin are presented as geometric means. Free concentrations of 25(OH)D and 1,25(OH)_2_D were calculated using published mathematical models [13] that include concentrations of total 25(OH)D, total 1,25(OH)_2_D, DBP and albumin. Group differences in baseline characteristics and early-morning fasted blood samples were tested by ANOVA with Scheffé post-hoc test or χ^2^ test. We made no adjustment for multiple testing.

Fourier regression was used to model diurnal variation as previously described [9], [14]. All concentration data were logged before analysis and data were modelled by country. The Fourier model uses two pairs of sine and cosine terms as independent predictors of the rhythm and a random effect to allow for individual intercepts and within-individual correlation of data from the multiple data points per individual. The significance of the rhythm was determined by testing the null hypothesis that all Fourier coefficients were zero. Different methods are available to measure and compare the amplitudes of cyclic rhythms [15]. We used a method that allows the assessment and comparison of the magnitude of the amplitude on a common scale. It was expressed as a mean (95% CI) coefficient of cyclic variation (CCV%), calculated as the square root of half the sum of the squared coefficients of the Fourier terms [15]. Differences in CCV% were compared between countries and between bound and free metabolites using the z-test. Country differences in 24 h means were tested by including country in a regression model of the plasma concentration of the analyte of interest against the sine and cosine parameters. Cross-correlation analysis on a per country basis was used to examine the relationship between variables and the strongest correlation, *r*, and lag time are presented. Statistical analysis and modelling was performed in Stata 13 SE (Stata Corp, TX, USA) and z-tests in Microsoft Excel.

## 3 Results

Participant characteristics and measured fasting, baseline biochemistry are shown in Table 1.

**Table 1:** Baseline participant characteristics and fasting, early-morning biochemistry.

	Britain (*n* 30)	Gambia (*n* 31)	China (*n* 30)
Age, y	65.5 (3.9)	66.1 (4.3)	64.4 (3.5)
Weight, kg	79.1 (73.3, 85.4)^G**,C**^	56.1 (53.1, 59.2)^B**,C*^	62.9 (60.1, 65.9)^B**,C*^
Height, m	1.72 (0.11)^G**,C*^	1.62 (0.10)^B**^	1.64 (0.08)^B*^
BMI, kg/m^2^	26.9 (25.0, 28.9)^G**,C*^	21.4 (10.5, 22.4)^B**^	23.0 (22.1, 24.0)^B*^
Sex, female/male	14/16	17/14	14/16
25(OH)D, nmol/L	37 (30,44)^G**^	59 (52, 65)^B**,C**^	32 (27, 38)^G**^
1,25(OH)_2_D, pmol/L	124 (110, 140)^G**^	186 (172, 201)^B**,C**^	123 (110, 137)^G**^
DBP, μmol/L	8.0 (7.5, 8.5)^[*n*29],G*^	7.2 (6.9, 7.6)^B*^	7.8 (7.4, 8.2)
Albumin, μmol/L	571 (557, 585)^G**,C**^	495 (481, 510)^B**,C**^	660 (646, 674)^G**,C**^
Free 25(OH), pmol/L	11.1 (9.1, 13.4) ^[*n*29],G**^	19.5 (17.5, 21.8)^B**,C**^	9.7 (8.2, 11.4)^G**^
Free 1,25(OH)_2_D, pmol/L	0.85 (0.76, 0.95)^[*n*29],G**^	1.42 (1.31, 1.54)^B**,C**^	0.83 (0.74, 0.93)^G**^

Values are mean (SD) or geometric mean (95%). Group differences were tested by one-way ANOVA with Scheffé post-hoc tests or Chi-squared test. Free 25(OH)D and free 1,25(OH)_2_D are calculated values. Superscripts indicate differences between countries (B-Britain; G-Gambia; C-China); * P < 0.05, ** P < 0.001

Gambians had significantly higher total 25(OH)D and total 1,25(OH)_2_D plasma concentrations and significantly lower albumin concentrations compared to the British and Chinese. DBP was slightly lower in Gambians but was only significant when compared with the British (Gambia vs China, P = 0.16). Albumin concentration was lowest in the Gambians and highest in the Chinese and differences were significant between all countries. Free 25(OH)D and free 1,25(OH)_2_D were higher in the Gambians compared to the other groups and there were no differences between the British and Chinese.

**Fig. 1:**
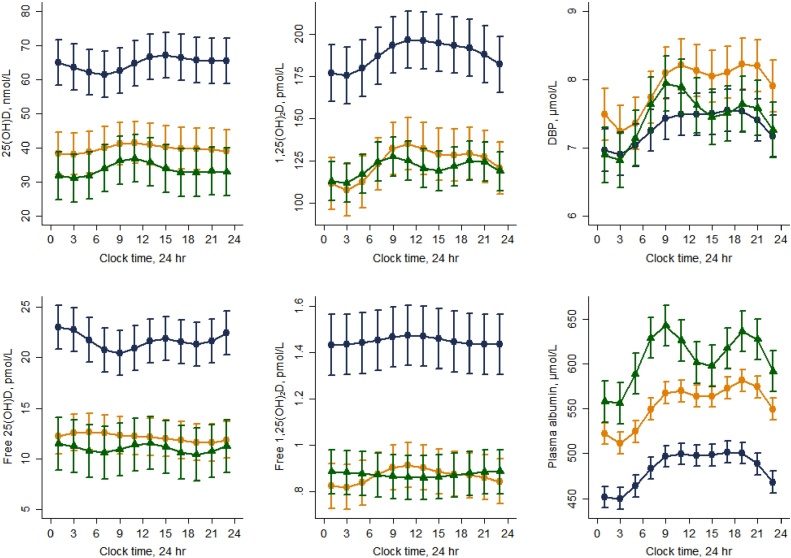
Vitamin D metabolites and their binding proteins exhibit significant diurnal rhythms that are attenuated for calculated free 1,25(OH)_2_D. Diurnal rhythms were assessed in British (orange circles), Gambian (blue diamonds) and Chinese (green triangles) older people. Lines represent the fitted values following Fourier regression and the error bars 2 * the upper and lower standard error. (For interpretation of the references to colour in this figure legend, the reader is referred to the web version of this article.)

Results of the Fourier regression are shown in Fig. 1 and Table 2. The 24 h mean concentrations derived from the DR analysis were generally similar to the fasting, baseline concentrations (within 0–15%) with vitamin D metabolite concentrations consistently higher in Gambians compared to the British and Chinese (Table 2 and Fig. 1). In contrast to the fasting values, 24 h mean concentrations of total 25(OH)D, DBP and albumin were higher in the British compared to the Chinese (Table 1, Table 2).

**Table 2:** Diurnal rhythm parameters.

	Britain (*n* 30)	Gambia (*n* 31)	China (*n* 30)
25(OH)D			
24 h mean, nmol/L	37 (31, 43)^G**,C*^	62 (56, 69)^B**,C**^	29 (25, 35)^B*,G**^
Rhythm significance, P	0.002	< 0.0001	< 0.0001
CCV%	2.2 (1.1, 3.4)^C*^	3.0 (2.1, 3.8)^C*^	5.7 (3.5, 7.9)^G*,B*^

1,25(OH)_2_D			
24 h mean, pmol/L	118 (105, 132)^G**^	183 (169, 198)^B**,C**^	116 (106, 127)^G**^
Rhythm significance, P	< 0.0001	< 0.0001	0.001
%CCV	7.1 (6.3, 8.0)^G**,C**^	4.4 (3.5, 5.4)^B**^	3.8 (2.6, 4.9)^B**^

DBP			
24 h mean, μmol/L	7.8 (7.5, 8.2)^G*,C*^	7.2 (7.0, 7.5)^B*,C*^	7.4 (7.0, 7.7)^B*,G*^
Rhythm significance, P	< 0.0001	0.001	< 0.0001
%CCV	4.5 (3.3, 5.6)	3.1 (1.9, 4.3)	4.8 (3.6, 6.0)

Albumin			
24 h mean, μmol/L	553 (544, 562)^G*,C*^	482 (472, 492)^B*,C*^	602 (589, 616)^B*,G*^
Rhythm significance, P	< 0.0001	< 0.001	< 0.0001
%CCV	4.1 (3.4, 4.7)	4.0 (3.4, 4.5)	4.7 (3.2, 6.2)

Free 25(OH)D			
24 h mean, pmol/L	11.2 (9.7, 13.0)^G**^	20.8 (18.9, 22.9)^B**,C**^	9.4 (7.9, 11.3)^G**^
Rhythm significance, P	0.008	0.005	0.1
%CCV	2.6 (1.2, 4.1)	3.0 (1.7, 4.4)	3.1 (0.5, 5.7)

Free 1,25(OH)_2_D			
24 h mean, pmol/L	0.83 (0.75, 0.92)^G**^	1.41 (1.30, 1.52)^B**,C**^	0.84 (0.76, 0.93)^G**^
Rhythm significance, P	< 0.0001	0.01	0.04
%CCV	3.2 (2.2, 4.2)^G*,C*^	1.4 (0.3, 2.6)^B*^	1.3 (0.1, 2.5)^C*^

Values are geometric mean (95% CI). The CCV% (coefficient of cyclic variation) is the standardized magnitude of the rhythm. Free 25(OH)D and free 1,25(OH)_2_D parameters are based on are calculated values. Superscripts indicate differences between countries (B-Britain; G-Gambia; C—China); * P < 0.05, ** P < 0.001.

A significant DR was present for all analytes in each country, with the exception of free 25(OH)D in the Chinese. Total 25(OH)D, total 1,25(OH)_2_D, DBP and albumin had lower values during the night and higher values during the day in all groups. The DR of free 1,25(OH)_2_D was attenuated compared to that of total 1,25(OH)_2_D as evidenced by the significantly lower CCV% (all P < 0.01). In contrast, the CCV%s between total 25(OH)D and free 25(OH)D were not different for any country (P > 0.05). There were no country differences in the magnitude of the rhythm (assessed by CCV%) for DBP, albumin or free 25(OH)D and there were no consistent country differences between the other metabolites.

In all countries, the strongest and most consistent cross correlation was between predicted values for DBP and albumin (all countries 0 h time lag, r 0.94–0.98, P < 0.0001 and DBP and total 1,25(OH)_2_D (all countries 0 h time lag, all r 0.95 P < 0.0001). The cross correlation between total 25(OH)D and DBP was also highly significant (r 0.84–0.92; all P < 0.0001) but the lag times were less consistent, varying between 0 and 3 h, compared to cross correlation between DBP and total 1,25(OH)_2_D. We examined the cross correlation between PTH and total and free 1,25(OH)_2_D in each country (all P < 0.0001) and found similar patterns for both total and free 1,25(OH)_2_D.

## 4 Discussion

We have shown significant diurnal rhythms (DR) in total 25(OH)D, total 1,25(OH)_2_D, DBP, albumin and the free concentrations of both 25(OH)D and 1,25(OH)_2_D. In this study, although the magnitude of the free 1,25(OH)_2_D DR was significant, it was attenuated compared to total 1,25(OH)_2_D as reflected by its smaller magnitude (CCV%). This suggests that total and free 1,25(OH)_2_D concentrations respond to variation in DBP concentration and that the free 1,25(OH)_2_D concentration is maintained within a relatively narrow range through known feed-back mechanisms such as upregulation of CYP24A1, which catabolizes 1,25(OH)_2_D. This is in accordance with the strict metabolic control of the circulating concentration of 1,25(OH)_2_D and the rapid feedback mechanism upon its activation of the VDR. There are reports of a DR of 1,25(OH)_2_D in some [9], [16] but not all human studies [17], [18], [19]. These differences may be due to the methods of analysis of the rhythm, sample size or the study population [16]. Only one study has reported a significant DR in the plasma concentrations of DBP, a study that showed no DRs in the calculated free-1,25(OH)_2_D index [16]. A single study has demonstrated a DR of 25(OH)D in humans [20].

Evidence of the potential influence of the DBP concentration on plasma of 1,25(OH)_2_D also comes from other human and animal studies. A significant positive association between plasma DBP and 1,25(OH)_2_D concentration has been reported in a cohorts in Denmark [21] and Sweden [4] and in pregnancy an increase in DBP is associated with an increase in 1,25(OH)_2_D [22]. In DBP-null mice, circulating total 1,25(OH)_2_D was only 1% of that in the wild-type but cellular uptake and biological activity were not compromised [23] potentially because free 1,25(OH)_2_D is regulated and thus was maintained within narrow ranges.

In contrast, there was no difference in the CCV% between total 25(OH)D and free 25(OH)D and the DR pattern of both total and free 25(OH)D poorly reflected that of the DRs of DBP and albumin. This indicates that free and total 25(OH)D do not respond as strongly as those of 1,25(OH)_2_D to changes in the plasma concentrations of its binding proteins, possibly because the concentration of free 25(OH)D is more dependent on 25(OH)D concentration than DBP concentration [24]. This is also consistent with the long plasma half-life of 25(OH)D (2–3 weeks) [25] and the limited metabolic control of the plasma concentration compared to 1,25(OH)_2_D that has a much shorter plasma half-life (∼3 d) [26]. This observation may be surprising given that, of all the vitamin D metabolites, 25(OH)D has the highest affinity for DBP.

The relationship between vitamin D metabolites and DBP and their free fractions may influence vitamin D availability, their half-lives and biological activity. Vitamin D metabolites are thought to be transported into cells predominantly by two mechanisms (1) DBP interaction and internalisation with the plasma membrane transporter protein, megalin, and (2) diffusion of the free metabolite across the cell membrane. In the kidney the megalin route is thought to be predominant but uptake of 25(OH)D into other cell types (e.g. monocytes [27]) is thought to be dependent on the diffusion of the free fraction. These and many other cell types express CYP27B1 and can produce 1,25(OH)_2_D for intra- or paracrine effects. Our understanding of the relative importance of these two mechanisms and their regulation in other tissue and cell types is in its infancy.

The absence of a pronounced DR of free 1,25(OH)_2_D is of interest in the context of the rhythmicity of PTH and earlier reported significant cross correlations between PTH and total 1,25(OH)_2_D [9]. These data may imply that PTH regulates the production of total 1,25(OH)_2_D but may not influence its free fraction. The latter may be predominantly controlled by feedback mechanisms upon VDR activation and/or the rate of 1,25(OH)_2_D internalisation and catabolism. There may be differences in the DRs of PTH and bone turnover markers in people with pathological conditions such as osteoporosis [28], [29] and diabetes [20] but this has so far not been shown for 1,25(OH)_2_D. Plasma concentrations of 1,25(OH)_2_D reflect the renal rather than the extra-renal production of 1,25(OH)_2_D. Other cell types which express the CYP27B1 and CYP24A1 may produce and catabolize 1,25(OH)_2_D intracellularly and thus may have an independent DR. Further investigation is needed to understand whether systemic or intracellular rhythmicity are involved in the regulation of the circadian rhythm of bone remodelling and other cell types, such as adipose-derived stem cells [30], [31].

DBP and albumin exhibited significant DRs similar to those reported in previous studies [16], [32], [33] and their patterns were similar for all three groups. Since these two molecules have very different half-lives (DBP ∼ 2-3 d [34]; albumin ∼ 20 d [35]) it is surprising that the DRs are similar and suggests that the DR in these plasma proteins may be related less to metabolism and more to other factors affecting their concentration. For example, circadian rhythms in glomerular filtration rate may affect and influence the daily rhythm of higher molecular weight proteins such as albumin and DBP [33]. Also hydration status, posture and nocturnal haemodilution (whereby blood volume is greater at night than in the daytime [16]) may alter the volume of distribution, or transfer and the distribution between interstitial and intravascular fluid compartments. Such a change in dilution volume and tissue distribution could produce variability in plasma DBP and albumin concentration leading to variability in total 1,25(OH)_2_D concentration and maintenance of free 1,25(OH)_2_D.

Fasting baseline and 24 h mean concentrations of total 25(OH)D and total 1,25(OH)_2_D were higher in Gambian compared to British and Chinese participants. Elevated 1,25(OH)_2_D in Gambians is well documented and is probably related to their low dietary calcium intake and high PTH concentration as well as potentially the supply of 25(OH)D [9], [11], [36]. Lower plasma albumin in black populations has been observed previously in some studies in the US [37] and in previous comparative studies in The Gambia [38]. The lower albumin concentrations in Gambians may be due to genetic factors, higher levels of inflammation or lower dietary protein intake. Slightly higher plasma albumin was reported in Asians in the US [39] but the reasons for the higher albumin in our Chinese group are unclear. Whist albumin circulates at much higher concentrations than DBP it’s contribution as a carrier of vitamin D metabolites is relatively minor due to a much lower binding affinity. Consequently, variation in calculated free 25(OH)D or 1,25(OH)_2_D concentration attributable to albumin is much lower than that of 25(OH)D, 1,25(OH)_2_D or DBP concentration. For DBP concentrations, large differences between populations were influenced by the assay used for quantification [40] and data generated using other assays and mass spectrometric-based methods suggest less variation in DBP concentration due to race or genotype [5]. The timing of the peak and nadir of the DRs of DBP were similar between groups despite differences in lifestyle, diet and vitamin D status [9]. This suggests that these DRs are influenced by factors in common between countries, including those discussed above, and potentially an endogenous circadian component. Known environmental and genetic differences between groups did not appear to have an influence on the observation that the DR magnitudes were different between free 1,25(OH)_2_D and total 1,25(OH)_2_D.

This exploratory study has some limitations. DRs were calculated based on a limited number of data points and although, as much as practicable, participants were asked to follow their normal routines, by the nature of these types of studies this was not entirely possible [9]. By their nature, invasive studies of this type are relatively small and this study was no exception. The study results for each group may therefore not be fully representative of their respective populations. The use of calculated free 25(OH)D and 1,25(OH)_2_D for assessing free concentrations of vitamin D metabolites may only be suitable for use in healthy populations. Where vitamin D metabolite or DBP concentrations are significantly altered due to physiological or pathophysiological conditions they may be less applicable [6], [25], [41]. We used a calculation that includes concentrations of 25(OH)D, 1,25(OH)_2_D, DBP and albumin but did not consider DBP genotype. DBP genotype, by affecting binding affinity for 25(OH)D, is suggested to affect the free 25(OH)D concentration [13]. However, other studies have shown that there are no differences in the affinity of 25(OH)D for DBP (summarised in [24]). Adjustment for DBP genotype would likely make only minor differences to the calculated free metabolite concentrations [13] and no difference to patterns within an individual.

In conclusion, we have shown significant DRs in vitamin D metabolite and DBP concentrations. These data show the importance of standardisation of the timing of sample collection in studies. The significant correlation between 1,25(OH)_2_D and DBP and lower CCV% for free 1,25(OH)_2_D compared to total 1,25(OH)_2_D suggests that 1,25(OH)_2_D may respond to changes in DBP possibly to maintain free 1,25(OH)_2_D concentrations. In contrast, the DRs of total and free 25(OH)D appeared to be less influenced by DBP, suggesting that DBP has comparatively less effect on 25(OH)D concentration.
